# Salience and default‐mode network connectivity during threat and safety processing in older adults

**DOI:** 10.1002/hbm.25199

**Published:** 2020-09-16

**Authors:** Lars Marstaller, Samuel Fynes‐Clinton, Hana Burianová, David C. Reutens

**Affiliations:** ^1^ Department of Psychology Bournemouth University Bournemouth UK; ^2^ Department of Psychology Swansea University Swansea UK; ^3^ Centre for Advanced Imaging University of Queensland Brisbane Australia; ^4^ Rotman Research Institute at Baycrest Toronto Ontario Canada

**Keywords:** ageing, conditioning, ICA, threat

## Abstract

The appropriate assessment of threat and safety is important for decision‐making but might be altered in old age due to neurobiological changes. The literature on threat and safety processing in older adults is sparse and it is unclear how healthy ageing affects the brain's functional networks associated with affective processing. We measured skin conductance responses as an indicator of sympathetic arousal and used functional magnetic resonance imaging and independent component analysis to compare young and older adults' functional connectivity in the default mode (DMN) and salience networks (SN) during a threat conditioning and extinction task. While our results provided evidence for differential threat processing in both groups, they also showed that functional connectivity within the SN – but not the DMN – was weaker during threat processing in older compared to young adults. This reduction of within‐network connectivity was accompanied by an age‐related decrease in low frequency spectral power in the SN and a reduction in inter‐network connectivity between the SN and DMN during threat and safety processing. Similarly, we found that skin conductance responses were generally lower in older compared to young adults. Our results are the first to demonstrate age‐related changes in brain activation during aversive conditioning and suggest that the ability to adaptively filter affective information is reduced in older adults.

## Graphical abstract

We measured skin conductance responses as an indicator of sympathetic arousal and used functional magnetic resonance imaging and independent component analysis to compare young and older adults’ functional connectivity in the default mode (DMN) and salience networks (SN) during a threat conditioning and extinction task. While our results provided evidence for differential threat processing in both groups, they also showed that functional connectivity within the SN – but not the DMN – was weaker during threat processing in older compared to young adults. Our results are the first to demonstrate age‐related changes in brain activation during aversive conditioning and suggest that the ability to adaptively filter affective information is reduced in older adults.

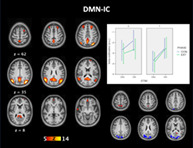

## 1 INTRODUCTION

Healthy ageing is associated with neurobiological changes in the brain's structural and functional organisation, which impact cognitive and affective functioning (Burianová et al., 2015; Grady, 2012; Razlighi et al., 2017). In particular, such age‐related changes might reduce an individual's ability to appropriately assess the emotional value of a stimulus, which might lead to adverse consequences during risky decision‐making, particularly in contexts of threat and safety (Huang, Wood, Berger, & Hanoch, 2015; Mikels, Cheung, Cone, & Gilovich, 2013; Weller, King, Figner, & Denburg, 2019). To identify age‐related changes in threat and safety processing, we used classical threat conditioning and compared behavioural, physiological, and neural responses in young and older adults.

Classical threat conditioning paradigms typically compare responses to conditioned stimuli (CS+) that have been repeatedly paired with an aversive unconditioned stimulus (US; e.g., electric shock, a loud sound, or loss of money) – and therefore come to represent threat – with responses to conditioned stimuli (CS‐) that have never been paired with a US and therefore come to represent safety. Threat and safety learning are most commonly assessed by measuring psychophysiological signals of arousal, such as the skin conductance response, which indicate the sympathetic nervous system's defensive response to direct, impending threat. Previous studies investigating threat conditioning in older adults have shown no age‐related differences in older adults' behavioural or psychophysiological differential responses to conditioned stimuli beyond a general reduction in arousal (Battaglia, Garofalo, & di Pellegrino, 2018; LaBar, Cook, Torpey, & Welsh‐Bohmer, 2004; Rosenbaum et al., 2015). However, the absence of behavioural and psychophysiological differences does not necessarily imply the absence of neural differences. A wealth of evidence supports the idea that the brains of older adults compensate for neural changes and cognitive decline by recruiting additional neural resources to achieve behavioural performance comparable to young adults (Burianová et al., 2015; Burianová, Lee, Grady, & Moscovitch, 2013; Morcom & Johnson, 2015; Reuter‐Lorenz & Lustig, 2005).

In younger adults, the neural activity during a classical threat conditioning paradigm has been shown to involve two patterns associated with threat and safety respectively (Fullana et al., 2016). The neural pattern underlying threat includes structures associated with interoception (such as the anterior insula and anterior cingulate cortex) and somatosensation (such as the secondary somatosensory cortex, thalamus, and cerebellum). In contrast, the brain activation underlying safety includes structures associated with stimulus‐independent processes in the cortical midline (such as dorsal anterior and ventromedial prefrontal cortices, posterior cingulate and retrosplenial cortices) and temporo‐parietal regions (such as the hippocampus, inferior and middle temporal cortices, inferior parietal cortex, and precuneus), as well as structures associated with somatosensation (such as the primary somatosensory cortex and posterior insula).

These neural correlates of threat and safety processing overlap with two central large‐scale brain networks, the salience network (SN) and default mode network (DMN; Fullana et al., 2016; Marstaller, Burianová, & Reutens, 2017). While the SN makes interoceptive predictions about homeostatically relevant (i.e., salient) internal or external stimuli (Seeley et al., 2007; Menon & Uddin, 2010; Uddin, 2015; Barrett & Simmons, 2015), the DMN provides and maintains context through the integration of abstract, transmodal information (Hasson, Chen, & Honey, 2015; Margulies et al., 2016; Marstaller et al., 2017; Murphy et al., 2018; Smith, Mitchell, & Duncan, 2018, 2019). Together, the SN and DMN are thought to form a unified allostatic system, in which cognitive context constrains interoceptive predictions about the physical consequences of stimuli and actions (Barrett, 2017; Kleckner et al., 2017). The interaction between the SN and DMN shapes how stimuli are evaluated, responded to, and experienced emotionally. The available evidence suggests that in the healthy young adult brain, the interaction between the SN and DMN is essential for the acquisition of evaluative responses to threat and safety. Yet, it is unclear whether and how the brain's affective responses are altered during healthy ageing.

Many neuroimaging studies have demonstrated that healthy ageing is associated with decreased functional connectivity in the SN and DMN (Marstaller, Williams, Rich, Savage, & Burianová, 2015; Putcha, Ross, Cronin‐Golomb, Janes, & Stern, 2016; Tomasi & Volkow, 2012) and that this loss of connectivity is associated with reduced performance on cognitive tasks, such as executive functioning or long‐term memory retrieval (Damoiseaux et al., 2008; Grady, St‐Laurent, & Burianová, 2015; Onoda, Ishihara, & Yamaguchi, 2012). Applied to threat and safety processing, this body of evidence suggests that age‐related changes in SN and DMN connectivity might be accompanied by changes in threat and safety processing.

Given the lack of neuroimaging studies, it is unclear whether previous behavioural findings are the result of neural compensation or reflect the absence of neural decline. Therefore, the goal of this study was to compare brain activity during threat and safety processing in healthy young and older adults using a classical threat conditioning and extinction paradigm. Based on the aforementioned studies, which show no behavioural differences between the two age groups, we expected to find comparable behavioural and psychophysiological responses in older and younger adults. Based on the evidence that healthy ageing is associated with reduced connectivity within the SN and DMN (He et al., 2014; Staffaroni et al., 2018), we further hypothesised that older adults would show weaker connectivity in those networks during threat and safety processing respectively. Finally, in line with the idea of compensation as behavioural equivalence through neural divergence, we expected that older adults would provide evidence for compensatory recruitment. Given that previous studies of healthy ageing most frequently report compensation in frontal regions (Burianová et al., 2013, 2015; Cabeza, Anderson, Locantore, & McIntosh, 2002; Reuter‐Lorenz & Lustig, 2005), we expected to find some evidence of compensatory recruitment in regions in medial frontal and anterior cingulate cortices of older compared to younger adults for threat and safety processing.

## 2 MATERIALS AND METHODS

### 2.1 Participants

Twenty‐eight older, right‐handed adults with normal or corrected to normal vision took part in the experiment after giving written consent. All participants self‐identified as native speakers of English. The study was approved by the Human Ethics Research Committee of the University of Queensland. Five data sets had to be excluded from the analysis because of excessive movement (two data sets), incidental findings (two data sets: macular degeneration and empty sella syndrome), and trauma, that was not disclosed during screening but after data acquisition (one data set: experience of terrorist attack). The final data set used in the analysis contains data from 23 participants (age M = 70.5 years, *SD* = 7.4 years, range = 59–83 years; 13 females; Mini Mental State Exam, M = 29.4, *SD* = 1.0; Folstein, Robins, & Helzer, 1983). As a control group, 23 data sets of young adults (age M = 26.2 years, *SD* = 3.5 years, range = 21–32 years; 12 females) were selected from a previously published study, which used the same experimental paradigm and acquired data using the same equipment and procedures (Marstaller et al., 2017).

### 2.2 Procedure

This experiment follows the procedure described in Marstaller et al. (2017). Participants took part in a partially reinforced, differential threat conditioning experiment with repeated conditioning and extinction blocks (A‐B‐A‐B paradigm). During each block, two visual stimuli, a black triangle or a black circle, served as conditional stimuli (CS) and were repeatedly presented in a randomised order. Each type of block (conditioning or extinction) was associated with a different background colour (blue or orange) and the association was randomised across participants. During the task, participants were asked to identify the stimuli by pressing one of two buttons with the second and third digits of their right hand. One of the two stimuli (CS+) was paired with an unconditional stimulus (US) during the first and third block (CON) but not the second and fourth block (EXT), while the other stimulus (CS‐) was never paired with the US.

Each block started with 15 sec of background presentation to allow the electro‐dermal response to settle and the participants to habituate. During each experimental block, 20 stimuli (10 CS+, 10 CS‐) were presented for 3 sec and followed by 15 sec of background. Two colours, blue and orange, served as backgrounds and were randomly associated with each experimental block. All stimuli were presented in a randomised order using Presentation software (v.20, Neurobehavioural Systems Ltd, https://www.neurobs.com/) and projected onto a screen, which could be viewed with a mirror attached to the head coil.

**FIGURE 1:**
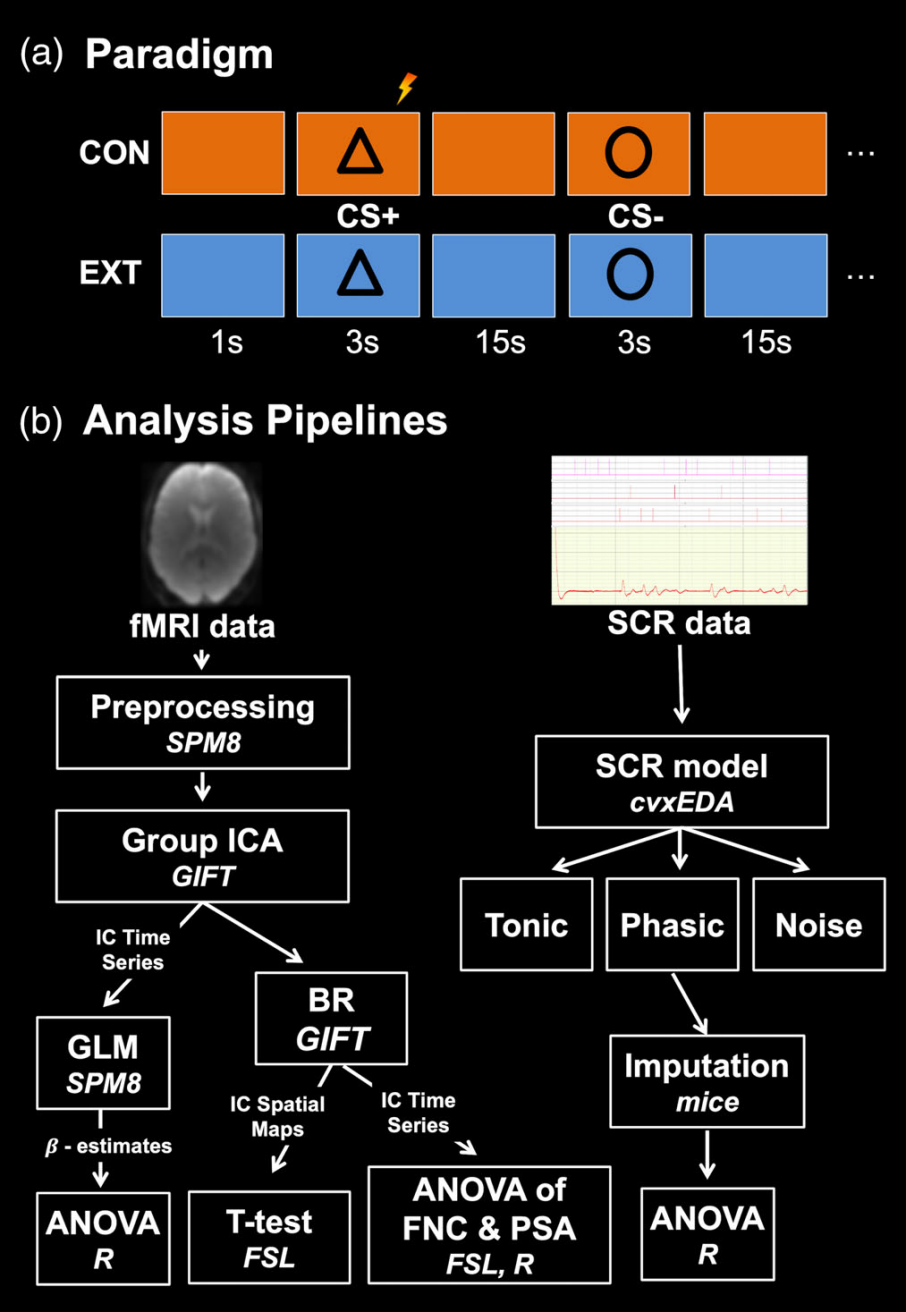
Description of the paradigm and analysis pipeline. (a) The figure shows the threat conditioning and extinction task, in which participants are presented with circles or triangles that are either reinforced (CS+) or not (CS‐) during the conditioning phase (CON), but never during the extinction phase (EXT). The different phases are marked by separate background colours (blue and orange). (b) The figure shows the analysis pipeline for fMRI and SCR data and includes the analysis steps and tools (in italics) used for each step. ICA, independent component analysis; IC, independent component; GLM, general linear model; BR, back‐reconstruction; FNC, functional network/internetwork connectivity; PSA, power spectrum analysis

Sixty percent of CS+ presentations co‐terminated with the US, which consisted of 50 ms transcutaneous electrical stimulation using two pre‐gelled carbon snap electrodes attached to the right wrist (EL508, Biopac Systems, Inc. https://www.biopac.com). Prior to scanning, the stimulation voltage was adjusted to individual tolerances following established procedures to ensure that stimulation was highly uncomfortable, but not painful (LaBar, Gatenby, Gore, LeDoux, & Phelps, 1998). Stimulation was administered using a STIMISOC isolator connected to a STM100C stimulator, which was controlled by a MP150 (Biopac Systems, Inc.). In addition to the procedure described in Marstaller et al. (2017), after each experimental block, older participants were asked to verbally provide a shock expectancy rating on a scale from 0 to 100 for the CS+ and CS‐ (see Figure 1a).

### 2.3 Psychophysiology acquisition and analysis

Electrodermal activity was recorded during neuroimaging and digitised at a sampling rate of 1 kHz with 16‐bit resolution using a Biopac MP150 system (Biopac Systems, Inc.). Electrodermal activity was measured on the medial phalanges of the index and middle fingers of the participant's left hand using pre‐gelled carbon snap electrodes (EL508, Biopac Systems, Inc.), amplified and low‐pass filtered (0.1 Hz) using an EDA100C amplifier (Biopac Systems, Inc.).

Data were downsampled to 1 kHz and analysed using a custom Python (v.3.6, https://www.python.org/) script using the Neurokit module (https://neurokit.readthedocs.io). The electrodermal data were analysed using the cvxEDA module (https://github.com/lciti/cvxEDA). cvxEDA uses a convex optimization approach to model the electrodermal activity of the recorded skin conductance response (SCR) and to derive estimates for phasic sudomotor nerve activity (SNA), tonic changes, and noise (Greco, Valenza, Lanata, Scilingo, & Citi, 2016). As an indicator of arousal, we computed the maximum SNA estimate for each non‐reinforced stimulus' SCR in the time window 1–4 sec post onset (Lockhardt, 1966). SNA values larger than 30 were considered unrealistic and excluded from the analysis resulting in 47% and 39% of missing data in the older and young adult group respectively. Missing *average SNA* values were imputed using multivariate imputation by chained equations as implemented in the “mice” package (v.3.4) in RStudio (v.1.2.1335) using predictive mean matching with 20 iterations of 100 imputations (Azur, Stuart, Frangakis, & Leaf, 2011; van Buuren & Groothuis‐Oudshoorn, 2011). One dataset in the older adult group was lost due to technical error. The final data for the skin conductance analysis included 22 older and 23 young adults.

### 2.4 MRI acquisition and preprocessing

Images were acquired with a Siemens Magnetom Trio 3T scanner and a 32‐channel head coil at the Centre for Advanced Imaging at the University of Queensland. For each participant, a T1‐weighted volumetric anatomical MRI was acquired with the following parameters: 176 slices sagittal acquisition MP2‐RAGE; 1 mm^3^ isotropic volume; repetition time (TR) = 4,000 msec; echo time (TE) = 2.89 msec; flip angle = 6°; FOV = 256 mm, GRAPPA acceleration factor = 3. Functional images were acquired using a T2*‐weighted echo‐planar image sequence with the following parameters: 45 slices; 2.5 × 2.5 × 2.7 mm voxel size; TR = 3,000 msec; TE = 30 msec; FOV = 192 mm; flip angle = 90°. Functional images were acquired in four runs, each corresponding to one experimental block.

Brain activation was assessed using the blood oxygenation level dependent (BOLD) effect (Ogawa, Lee, Kay, & Tank, 1990). For functional analysis, T2*‐weighted images were preprocessed with Statistical Parametric Mapping software (SPM8; http://www.fil.ion.ucl.ac.uk/spm). Images were realigned to the mean image for head‐motion correction and then spatially normalised into standard stereotaxic space with a voxel size of 2 mm^3^ (Montreal Neurological Institute template) using segmented white and grey matter T1 maps. Head movement and rotation in the three dimensions did not exceed 2 mm or 2° and no dataset had to be excluded from analysis. Finally, the functional images were spatially smoothed with an 8‐mm full width half maximum Gaussian kernel.

### 2.5 Independent component analysis

Following preprocessing, functional networks were identified with group independent component analysis (ICA) using the Group ICA of fMRI Toolbox (GIFT; http://mialab.mrn.org/software/gift/index.html; see Figure 1b). ICA is a method of blind source separation, which identifies source signals (indepentend components) in the fMRI data by maximising the signals' statistical independence. The resulting independent components (ICs) are defined as functional networks, in which neural activity operates in concert to generate a statistically independent signal. Each IC consists of a timecourse and a 3D map. The 3D map indicates the spatial extent of the network while the timecouse indicates the strength of the network signal (i.e., functional connectivity) in the data across time. To assess task‐relatedness, each IC's timecourse can be analysed with a general linear model in the same manner applied in mass‐univariate analysis.

Individual images were first normalised to their mean intensity and then concatenated across time. The optimal number of ICs was estimated to be 51 using the minimum description length algorithm (Li, Adali, & Calhoun, 2007). After data reduction with principal component analysis, 51 ICs were identified using the infomax algorithm (Bell & Sejnowski, 1995). To estimate the stability of ICs, this analysis was repeated 50 times using ICASSO (Hirnberg, Hyvärinen, & Esposito, 2004). Only those ICs with a stability index larger than 0.95 were selected for further analysis. Finally, back‐reconstruction was applied to estimate the spatial maps and time courses of each IC for each participant using dual regression (Calhoun, Adali, Pearlson, & Pekar, 2001).

Next, two ICs of interest were identified based on their overlap with the SN and DMN target networks identified in Laird et al. (2011) as ICNs 4 and 13. Overlap between target networks and ICs was calculated using the spatial involvement measure implemented in ICN_atlas (Kozák, van Graan, Chaudhary, Szabó, & Lemieux, 2017). The selected ICs will be referred to as salience network component (SN‐IC), and default‐mode network component (DMN‐IC) in the remainder of the report.

To identify differences in the task‐relatedness of ICs, a general linear model was fitted to each IC's time course. Subject‐specific regressors for CS+ and CS‐ were created for each of four imaging runs in SPM8 using convolution of a canonical hemodynamic response function with stick functions at the stimulus onsets. The beta‐estimates were compared using a 2 × 2 × 2 analysis of variance (ANOVA) with the within‐subjects factors stimulus (CS+, CS‐) and phase (conditioning, extinction), and the between‐subjects factor group (young, older) separately for each IC of interest.

**FIGURE 2:**
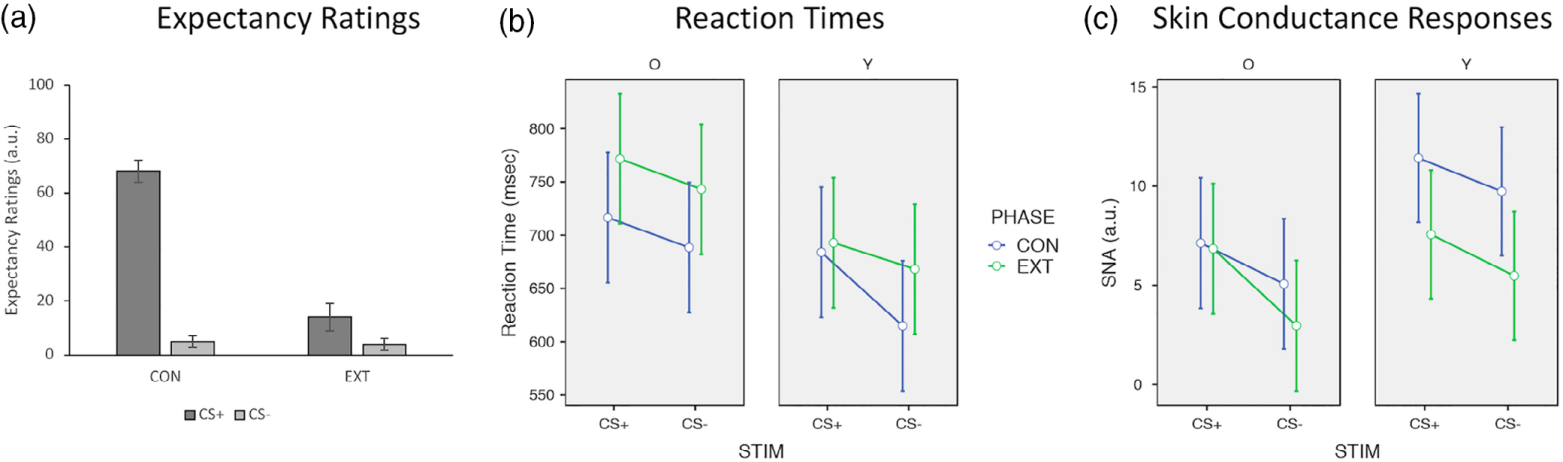
Behavioural and psychophysiological responses. The figure shows the means (and 95% confidence intervals) for expectancy ratings (a), response times (b), and the phasic component of the skin conductance response (c) for each combination of stimulus, phase, and group. CON, conditioning phase; EXT, extinction phase; STIM, stimulus; O, older adults; Y, young adults; SNA, sudomotor nerve activity

To identify group differences in network recruitment as well as recruitment of potentially compensatory areas in older adults, we also conducted non‐parametric tests based on the t‐statistics for comparisons of each IC of interest between young and older adults. For each session, back‐reconstructed individual maps of the SN‐IC and DMN‐IC were compared using FSL's randomise (FMRIB Software Library, v 5.0.10; https://fsl.fmrib.ox.ac.uk; Winkler, Ridgway, Webster, Smith, & Nichols, 2014) with 5,000 permutations and corrected for multiple comparison on the cluster level using family‐wise error correction (see Figure 2 for results, maps are thresholded at *p* < .001 FWE).

Finally, in order to assess the interaction between the SN‐IC and DMN‐IC, we computed the functional network connectivity as the correlation between the two networks' time courses for each subject's back‐reconstructed time course (Jafri, Pearlson, Stevens, & Calhoun, 2008). We further assessed the normalised spectral power in each back‐reconstructed time course across six frequency bins (Garrity et al., 2007). Both measures, functional network connectivity and spectral power, were computed using GIFT and compared between groups for each experimental phase separately.

## 3 RESULTS

### 3.1 Expectancy ratings

Older adults rated their expectancy of receiving a US following the CS+ (*M*_cond_ = 68.13, *SD*_cond_ = 21.0; *M*_ext_ = 13.98, *SD*_ext_ = 26.0) and the CS‐ (*M*_cond_ = 4.93, *SD*_cond_ = 11.1; *M*_ext_ = 3.85, *SD*_ext_ = 11.4) following each phase. Two paired *t*‐tests comparing expectancy ratings for the CS+ and CS‐ following conditioning and extinction showed that after conditioning older adults were correctly expecting the US following CS+ but not CS‐presentations (*t*[26] = 12.77, *p* < .001, *d* = 2.457) whereas their expectancy ratings did not differ significantly between conditioned stimuli after extinction (*t*[26] = 1.97, *p* = .06, *d* = .378; see Figure 2a).

### 3.2 Response times

A 2x2x2 analysis of variance of response times with within‐subjects factors stimulus (CS+, CS‐) and phase (conditioning, extinction), and between‐subjects factor group (younger, older adults) resulted in significant main effects of stimulus (*F*(1,43) = 6.7, *p* = .01, *η*_*p*_ ^2^ = .133) and phase (*F*(1,43) = 6.5, *p* = .01, *η*_*p*_ ^2^ = .129). The factor group almost met the significance criterion (*F*(1,43) = 3.5, *p* = .07, *η*_*p*_ ^2^ = .073). No interactions were significant (all *p* > .05). Together, these results show that participants responded faster during conditioning than during extinction but slower in response to CS+ than to CS‐ (see Figure 2b).

### 3.3 Skin conductance responses

A 2x2x2 analysis of variance of SNA estimates with within‐subjects factors stimulus (CS+, CS‐) and phase (conditioning, extinction), and between‐subjects factor group (younger, older adults) resulted in a significant main effects of group (*F*(1,43) = 5.1, *p* = .03, *η*_*p*_ ^2^ = .107) and stimulus (*F*(1,43) = 5.9, *p* = .02, *η*_*p*_ ^2^ = .12). The factor phase almost met the significance criterion (*F*(1,43) = 3.5, *p* = .07, *η*_*p*_ ^2^ = .08). No interactions were significant (all *p* > .05). Together, these results show that young adults produced higher overall skin conductance responses and provide evidence for successful differential conditioning in both groups (see Figure 2c).

### 3.4 Independent component analysis

The 2x2x2 ANOVA of the beta‐estimates for the DMN‐IC showed a significant main effect of stimulus (*F*(1,54) = 20.7, *p* < .001, *η*_*p*_ ^2^ = .277). No other effects or interactions were significant (all *p* > .05; see Figure 2a). A follow‐up t‐test showed that connectivity in the DMN‐IC was significantly lower in response to the CS+ than the CS‐ irrespective of phase or group (*t*[54] = 4.6, *p* < .001).

The 2x2x2 ANOVA of the beta‐estimates for the SN‐IC showed significant main effects of stimulus (*F*(1,54) = 5.7, *p* = .02, *η*_*p*_ ^2^ = .096) and group (*F*(1,54) = 4.9, *p* = .03, *η*_*p*_ ^2^ = .082). The factor phase almost met the significance criterion (*F*(1,54) = 3.1, *p* = .09, *η*_*p*_ ^2^ = .054). No other effects or interactions were significant (all *p* > .05; see Figure 2b). Follow‐up t‐tests showed that young adults engaged the SN‐IC more strongly than older adults (*t*[54] = 2.2, *p* = .03) and that both groups showed greater SN‐IC connectivity in response to the CS+ compared to the CS‐ (*t*[54] = 2.4, *p* = .02).

**FIGURE 3:**
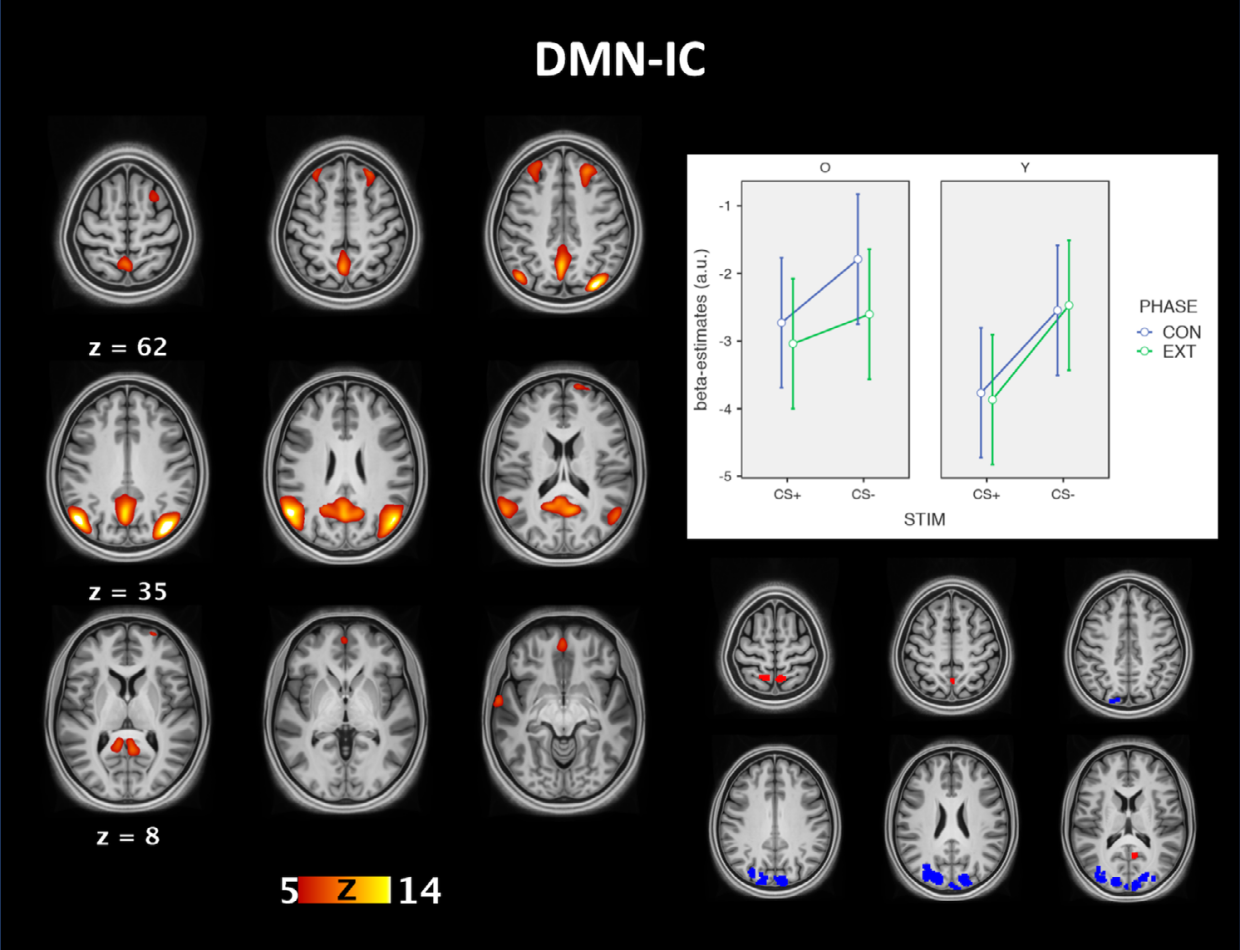
Group‐ICA results of the default mode network. The figure shows the connectivity pattern best matching the default mode network on the left. The graphs on the right show the mean (95% confidence intervals) beta‐estimates of the IC time courses for each combination of stimulus, phase, and group. The brain image on the bottom right shows the results of the voxel‐wise comparison of the back‐reconstructed connectivity maps between younger (red) and older adults (blue) (thresholded at *p* < .001 FWE). CON, conditioning phase; EXT, extinction phase; STIM, stimulus; O, older adults; Y, young adults

**FIGURE 4:**
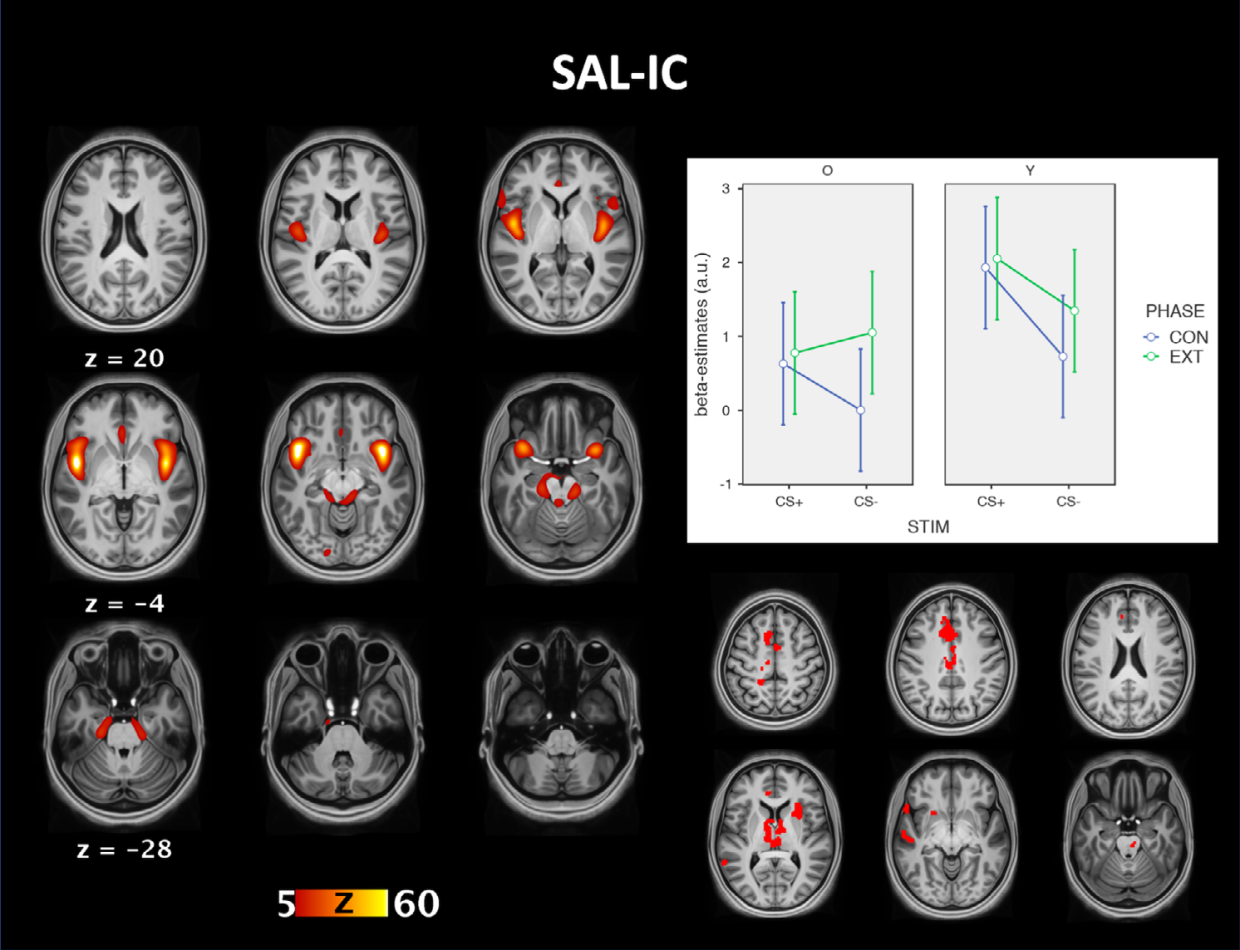
Group‐ICA results of the salience network. The figure shows the connectivity pattern best matching the salience network on the left. The graphs on the right show the mean (95% confidence intervals) beta‐estimates of the IC time courses for each combination of stimulus, phase, and group. The brain image on the bottom right shows the results of the voxel‐wise comparison of the back‐reconstructed connectivity maps between younger (red) and older adults (blue) (thresholded at *p* < .001 FWE). CON, conditioning phase; EXT, extinction phase; STIM, stimulus; O, older adults; Y, young adults

The group comparison of individual back‐reconstructed components revealed stronger functional connectivity of the DMN‐IC with occipital cortex in older adults and posterior cingulate cortex and precuneus in young adults. The group comparison further showed stronger functional connectivity of the SN‐IC with the anterior cingulate cortex, insula, and thalamus in young compared to older adults (see Figures 3 and 4).

A 2 × 2 ANOVA of functional network connectivity between the DMN‐IC and the SN‐IC with within‐subjects factor phase (conditioning, extinction) and between‐subjects factor group (younger, older adults) resulted in a significant main effect of group (*F*(1,45) = 42.5, *p* < .001, *η*_*p*_ ^2^ = .321). The factor phase almost met the significance criterion (*F*(1,45) = 3.9, *p* = .05, *η*_*p*_ ^2^ = .041). No other effects or interactions were significant (all *p* > .05). The mean correlation between the DMN‐IC and SN‐IC was −.25 (*SD* = .26) during the conditioning and − .35 (*SD* = .3) during the extinction phases for older adults compared to −.59 (*SD* = .26) during the conditioning and − .61 (*SD* = .25) during the extinction phases for younger adults).

Two‐sided t‐tests (Bonferroni adjusted p‐values for multiple comparisons) of the spectral power across time bins for each network further revealed significantly higher power in the SN‐IC (but not the DMN‐IC) for younger, compared to older adults, in the low frequency bins during conditioning (0–0.08 Hz: *t*(44) = 3.9, *p* = .009; 0.08–0.16 Hz: *t*(44) = 4.1, *p* = .005) and extinction (0–0.08 Hz: *t*(44) = 3.5, *p* = .03; 0.08–0.16 Hz: *t*(44) = −3.1, *p* = .13) phases. The results further show significantly higher power in both networks across both experimental phases for older, compared to younger, adults in the high frequency bins (> .16 Hz, all *p* < .05).

## 4 DISCUSSION

The aim of this study was to investigate age‐related differences in behavioural, psychophysiological, and neural responses to threat and safety, as well as to test for evidence of compensatory recruitment in older adults. All three types of data provided evidence of differential responding to threat and safety across both age groups. However, in contrast to young adults, older adults showed a general reduction in skin conductance responses and in SN connectivity irrespective of stimulus or experimental phase. Our results further showed age‐related changes in the SN, but not the DMN, including reduced connectivity in the anterior cingulate cortex, insula, and thalamus, and reduced power in the low frequency spectrum in older, compared to younger, adults. In addition, our results demonstrate that connectivity in the SN and DMN is less strongly anticorrelated in older, compared to younger, adults. Our results provide no evidence of compensatory activity in older adults.

The psychophysiological pattern of our results is in line with findings of previous studies, which report overall reduced skin conductance responses in older compared to young adults (LaBar et al., 2004; Rosenbaum et al., 2015). LaBar et al. (2004) further showed that skin conductance responses in older adults are dependent on contingency awareness. Post‐run expectancy ratings suggest that all older participants in this study were aware of the differential nature of the stimulus contingencies. This result suggests that contingency awareness might not be the only reason for the reduction in skin conductance responses in older adults. However, a large proportion of our electrodermal data was missing and had to be imputed. Hence, while showing the same pattern found previously, our results do not provide strong evidence against the importance of contingency awareness. Instead, our results highlight the need for further studies investigating the impact of contingency awareness on skin conductance responding during threat conditioning in older adults.

One potential explanation for the reduction in skin conductance responses is based on our fMRI data. The neuroimaging results demonstrate reduced task‐related connectivity in the SN in older adults; in particular, a reduction in the connectivity between the thalamus and the anterior cingulate cortex. Previous studies have established that the anterior insula is engaged in the perception and anticipation of salient stimuli whereas the anterior cingulate cortex is more strongly associated with behavioural adaptation (Ham, Leff, de Boissezon, Joffe, & Sharp, 2013; Holtz, Pané‐Farré, Wendt, Lotze, & Hamm, 2012; Lovero, Simmons, Aron, & Paulus, 2009; Menon & Uddin, 2010). In addition, research shows that SN connectivity modulates the degree to which autonomic responses are consciously experienced as arousal (Medford & Critchley, 2010; Xia, Touroutoglou, Quigley, Feldman Barrett, & Dickerson, 2017). Given this role of the SN in predicting and experiencing salient afferent inputs, our results suggest that older adults might prepare less for the US and, therefore, show a reduced sympathetic response to the CS. This interpretation receives further support from findings which show that ageing is associated with a shift from proactive to reactive cognitive control (Paxton, Barch, Racine, & Braver, 2008), and a reduction in attentional orienting (Bollinger, Rubens, Masangkay, Kalkstein, & Gazzaley, 2011; Hämmerer, Li, Müller, & Lindenberger, 2010), both of which have been shown to be related to the SN (Dosenbach et al., 2006; Nelson et al., 2010).

Interestingly, our results do not show a significant group difference in task‐related DMN connectivity. Previous studies generally report an inability of older adults to decrease activity in the DMN during externally directed tasks (Brown, Hakun, Zhu, Johnson, & Gold, 2015; Grady, Grigg, & Ng, 2012; Park, Polk, Hebrank, & Jenkins, 2010; Sambataro et al., 2010; Staffaroni et al., 2018). While our data show stronger decreases in DMN connectivity for young compared to older adults, the difference was not significant. However, our results do show significantly lower decreases in DMN connectivity in response to safety (CS‐) compared to threat stimuli (CS+). These results are in line with previous findings that young adults engage the DMN during safety processing (Marstaller et al., 2017; Zidda et al., 2018).

Finally, our results show that in older adults, connectivity within the DMN is significantly less anticorrelated with connectivity within the SN. In addition, our results show that low frequency BOLD‐related power in functional connectivity is reduced in older, compared to young, adults in the SN, but not the DMN. Together, these findings suggest that during threat and safety responding older adults do not engage the SN to the same degree as young adults and that this lower SN activity is not due to the DMN or the interaction between the DMN and SN.

Taken together, our results highlight important age‐related changes in threat and safety processing. A central idea in current cognitive neuroscience is that prediction of afferent signals is central to the brain's computational architecture (Bubic, von Cramon, & Schubotz, 2010; Hoemann, Gendron, & Barrett, 2017). In the context of threat and safety processing, the predictive framework suggests that the anticipation of affective values depends on interoceptive and visceromotor sensory predictions computed using a hierarchical, internal model of the body, which is constantly updated via the resulting prediction errors (Barrett, 2017; Barrett & Finlay, 2018; Barrett & Simmons, 2015; Kleckner et al., 2017). In particular, research increasingly suggests that the DMN constitutes the central backbone of such an internal model, whereas the SN acts as a filter that weighs prediction errors based on the prediction errors' relevance for allostasis within the current context (Barrett, 2017; Hasson et al., 2015; Margulies et al., 2016). Following this view, our findings that older adults under‐activate the SN relative to young adults during threat processing can be interpreted as a reduction of their ability to use prediction errors to adjust the internal predictive model to the current context. This age‐related reduction in the ability to adaptively filter prediction errors and to adjust the internal predictive model might then lead to a reduction in the processing of aversive and appetitive stimuli as well as to reduction in cognitive flexibility (Braver et al., 2001; Chowdhury et al., 2013; Eppinger, Hämmerer, & Li, 2011; La Corte et al., 2016; Onoda et al., 2012; Radulescu, Daniel, & Niv, 2016; Touroutoglou, Zhang, Andreano, Dickerson, & Barrett, 2018; van de Vijver, Ridderinkhof, & de Wit, 2015). Ultimately, these changes in SN connectivity might be caused by neurochemical changes in dopamine and serotonin levels, which affect both SN and DMN activation and play a central role in appetitive and aversive prediction error processing (Chowdhury et al., 2013; Conio et al., 2019; Dang, Donde, Madison, O'Neil, & Jagust, 2012; Li & Rieckmann, 2014; Nagano‐Saito, Liu, Doyon, & Dagher, 2009; Pignatelli & Bonci, 2015). Finally, the reduced ability of older adults' brains to adapt to fluctuating affective values in the environment could potentially explain the altered emotional responses commonly observed in older adults, such as the positivity effect (Mather & Carstensen, 2005; Reed, Chan, & Mikels, 2014).

In summary, our results are the first to show age‐related differences in brain activation during aversive conditioning. In particular, our findings highlight the role of the SN in the processing of aversive prediction errors and sympathetic nervous system responses. However, this study can only provide a starting point and further research into the ultimate causes of age‐related changes in SN connectivity, prediction error processing, and aversive conditioning are necessary.

## CONFLICT OF INTERESTS

The authors declare no conflicts of interest.

## Data Availability

Data Availability Statement: Data are available from the corresponding author on request.
